# The Carboxy Terminal Region on Spike Protein of Porcine Epidemic Diarrhea Virus (PEDV) Is Important for Evaluating Neutralizing Activity

**DOI:** 10.3390/pathogens10060683

**Published:** 2021-05-31

**Authors:** Ki-Jong Kang, Dong-Hwan Kim, Eui-Ju Hong, Hyun-Jin Shin

**Affiliations:** 1 Laboratory of Infectious Diseases, College of Veterinary Medicine, Chungnam National University, Daejon 34134, Korea; daesungman@hanmai.net (K.-J.K.); shin0089@gmail.com (D.-H.K.); ejhong@cnu.ac.kr (E.-J.H.); 2 Research Institute of Veterinary Medicine, Chungnam National University, Yuseong-gu, Daejon 34134, Korea

**Keywords:** porcine epidemic diarrhea virus, spike protein, carboxy terminal, neutralizing activity

## Abstract

In this study, we evaluated 62 sow sera samples from PED-vaccinated sows to compare the serum neutralizing test (SNT) and enzyme-linked immunosorbent assay (ELISA). We performed protein ELISA (pELISA) using fragments of spike proteins S1, S2, S3 and entire nucleocapsid proteins, and found a correlation between the SNT and ELISA in PEDV-vaccinated sera. Sera with higher neutralizing activity showed higher titers of IgG. In the antibody profiling, the neutralizing activities are correlated with the levels of the spike antibody, especially the S3 region. We confirmed that the carboxy-terminal region, including the endodomain of the S protein, induced stronger neutralizing activity than the ectodomain. This region of the S protein could be useful for evaluating PED vaccine efficacy, and it is a strong neutralizing epitope of PEDV. The S3 protein could be useful for evaluating PED vaccine efficacy, and it is a strong neutralizing epitope of PEDV.

## 1. Introduction

PEDV is an enteric pathogen affecting pigs of all ages that causes acute watery diarrhea among suckling piglets and weight loss. Clinical signs in infected piglets are mainly dehydration by diarrhea and some piglets showing vomiting but not in all cases [1]. PED is usually fatal in suckling piglets, morbidity and mortality are comparably high [1]. In older pigs, such as weaned pigs and sows, morbidity is high but the mortality rate is low [2]. After first identified in the UK in the 1970s, disease spread to European countries [3]. In 2010, new variants with high morbidity were reported in China, new reports with the same strains were from many other countries such as US, Canada, Mexico, Brazil and so on [4].

The role of each structural protein of PEDV has been studied based on other coronaviruses. The role of nucleocapsid (N) protein reported that it bound to the viral genomic RNA and is also involved in interferon inhibition in infected cells [2,5]. Spike protein (S) in PEDV has the most critical role in its infection, especially in very early stages. It is involved in virus entry for binding with its receptor and decision factors for tissue tropism [6]. As the most important role in entry, therefore, it is the main target of neutralizing antibodies [7]. Amino acids variation on the spike protein creates variants and decision factors for their grouping, G1 and G2 [8].

A diagnosis of PED should be performed in laboratory not only by clinical signs or postmortem because many other swine pathogens also induce similar diseases with PED such as transmissible gastroenteritis (TGE), colibacillosis, Salmonella and Rota virus infection [9,10]. Many techniques have been used for the detection of PEDV, including virus isolation, immunofluorescence assay (IFA), polymerase chain reaction (PCR), immunohistochemistry (IHC), and ELISA [1,10,11,12,13,14,15,16]. Among these diagnostic methods, the RT-PCR method is a specific and sensitive diagnostic tool for the detection of PEDV from suspected tissue or fecal sample [17]. EM is used to detect viral particles in fecal material with diarrhea, but EM is not a sensitive technique [18]. Although, SNT is time-consuming with serial dilution of serum samples but SN test is important for vaccine efficacy evaluation since it confirms real protection values [11,14,16]. Mostly, whole virus has been used as coating antigen in ELISA but as the alternative choice, protein-based ELISA was also developed [11]. For preparation of whole virus coating antigen, cell culture, collection, centrifugation and titration are required, and it is a very time-consuming process. To solve this issue, protein-based ELISA has been developed [19,20]. The advantage of protein ELISA is that it is a much easier, cheaper and faster process for preparation of coating antigen. Lirola et al. reported that ELISA with structural proteins of PEDV, S1 provided important results for sensitivity and differentiation with other swine enteric pathogens [20]. Not only that, they also found S1 antibodies were the most important detection marker for early PEDV infection as antibodies generated against S1 were generated faster than other structural proteins. As detection of PED in the field only by clinical signs and/or pathology is hard to confirm, whole virus ELISA has been used to confirm in laboratory. PEDV is an enteric pathogen and serum antibodies are not always confirmed protection; rather, it is confirmation of individuals that had contact with PEDV [18]. Careful studies for antibody evaluation are required in PED studies.

**Figure 1:**
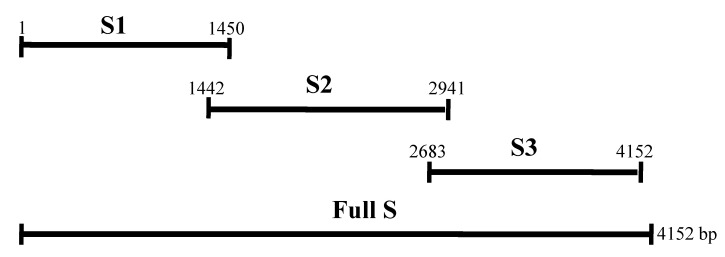
Schematic diagram of complete spike protein and locations of fragments S1, S2, and S3. Schematic diagrammatic representation of the complete spike protein and the corresponding locations of fragments S1, S2, and S3. The S1 and S2 regions are located in the ectodomain of the spike protein and the S3 region is located in the endodomain.

As there is no reported correlation between SN titer and ELISA values in PEDV infection, this study covers the screening of vaccinated pig samples on the basis of SNT and ELISA. Additionally, the utilization of protein ELISA using fragments of spike protein and nucleocapsid protein was evaluated (Figure 1).

## 2. Results

### 2.1. Expression of the Recombinant Spike and N Proteins

**Figure 2:**
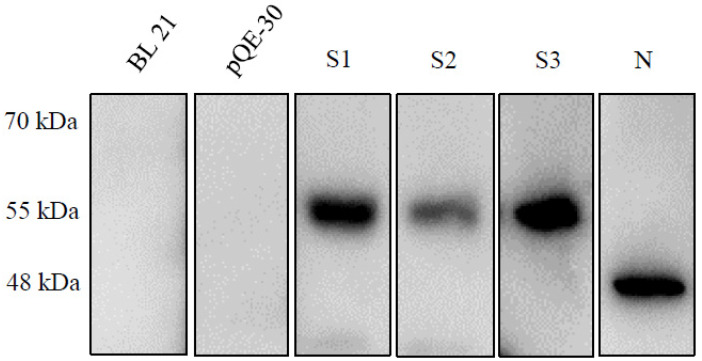
Western blot analysis of expression of spike fragments S1, S2, and S3 and nucleocapsid proteins in *E. coli.* The molecular weights of the S1, S2, S3 and N proteins were consistent with the predicted sizes of 55, 57, 57 and 48 kDa, respectively.

We confirmed the S1, S2, S3 and N proteins by Western blotting. The molecular weights of the S1, S2, S3 and N proteins were consistent with the predicted sizes of 55, 57, 57 and 48 kDa, respectively. As shown in Figure 2, the expression level of S3 was dominant, similar to N expression but slightly larger. S1 expression was also high but still lower than that of S3 or N. S2 expression was the lowest, much lower than that of the other proteins.

### 2.2. Serum Neutralization Test (SNT)

**Figure 3:**
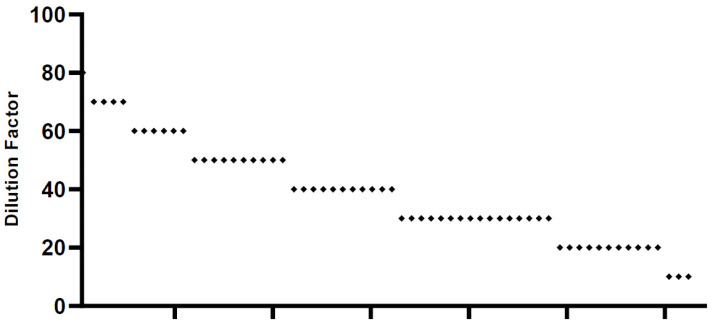
SNT results. SNT results (*n* = 62) were spotted based on dilution factors. We tested each sera sample and its neutralizing dilution factors are plotted individually. Dots are number of sera samples in each dilution factor.

We screened all 62 sera samples collected. We found that the SNT values in each serum sample were highly variable, between 10 and 80 (Figure 3). Only one typical serum sample showed the highest neutralizing activity up to 80 times dilution. Four samples showed activity up to 70 times dilution, 6 were active up to 60 times dilution, 10 were active up to 50 times dilution, 11 were active up to 40 times dilution, 16 were active up to 30 times dilution, and 8 samples had similar activity to that of 6 negative sera samples, which were nonvaccinated sera. Based on the SNT data, we categorized the patients into three groups: high, medium and low. The sera that had neutralizing activity at higher than 60 times dilution were included in the high category, those with activity at 30–60 dilutions were classified in the middle, and those with activity under 20 dilutions were included in the low group. Following this categorization, there were 11 samples in the high group, 43 in the middle group, and 8 in the low group.

### 2.3. Enzyme-Linked Immunosorbent Assay (ELISA)

**Figure 4:**
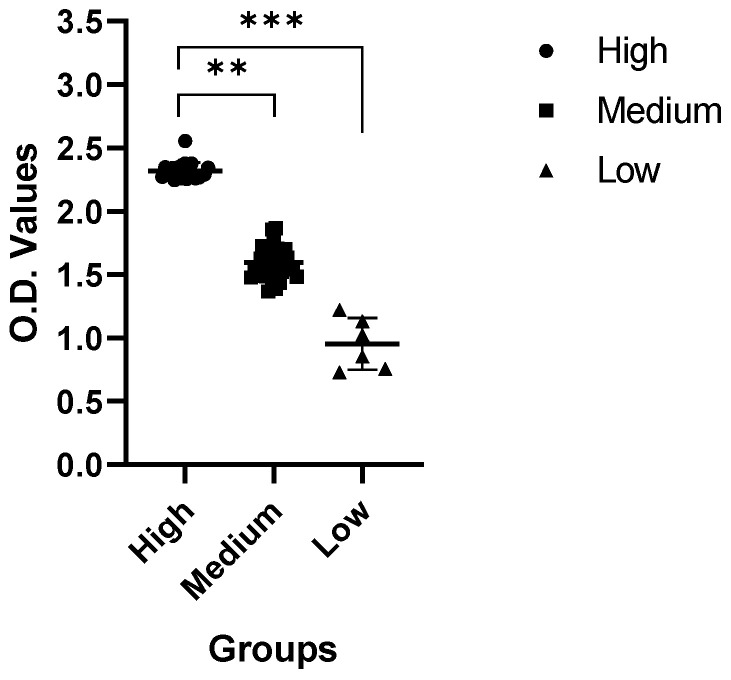
ELISA results. ELISA results are for whole PEDV as the coating antigen. IgG titer was between 2.253 and 2.362 OD value in the high group. The IgG titer was 1.478–1.865 in the middle group and 0.727–1.131 in the low group.

After categorization of the sera samples based on the SNT activity, we investigated the IgG levels in the samples. We evaluated and compared IgG levels in five sera samples from each category group. As shown in Figure 4, the IgG titer was between 2.253 and 2.362 OD value in the high group. The IgG titer was 1.478–1.865 in the middle group and 0.727–1.131 in the low group. Correlated with the SNT results, samples with higher SNT titers showed lower IgG titers, and samples with lower SNT titers showed higher IgG titers (Figure 4). Our results confirm that there was a clear correlation between the SNT and ELISA titer.

### 2.4. Protein ELISA (pELISA)

Next, we performed pELISA evaluation to determine the specific domain on the spike where neutralizing activity was generated in PEDV-vaccinated sera. We evaluated and compared the OD values from reactions with different proteins, such as the coating antigen, S1, S2, S3, and nucleocapsid proteins.

**Figure 5:**
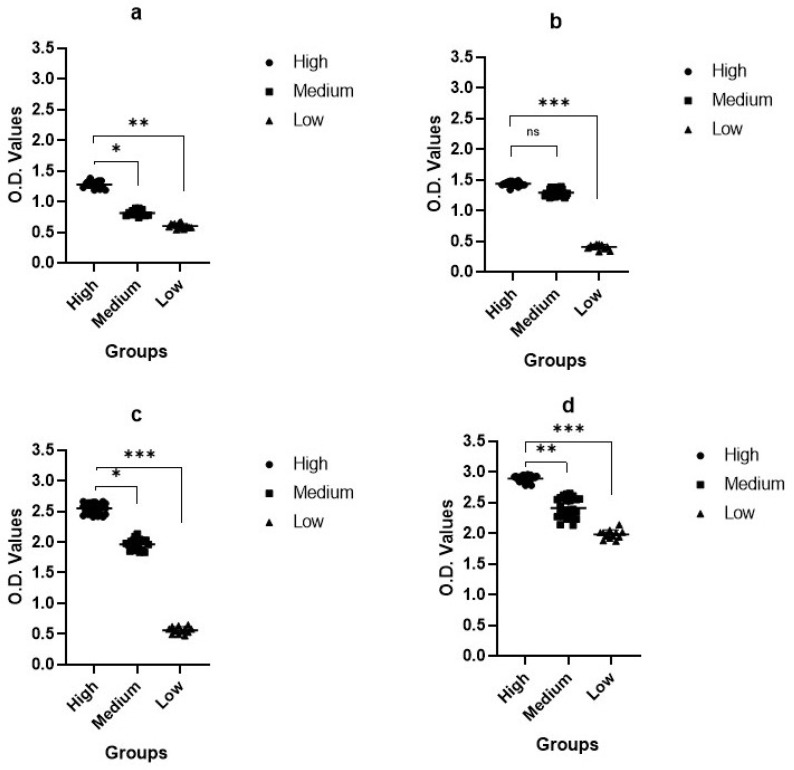
pELISA results. pELISA results are for S1, S2, S3, and nucleocapsid proteins as the coating antigen. (**a**) S1 coating antigen, (**b**) S2 coating antigen, (**c**) S3 coating antigen, (**d**) N coating antigen. We compared OD values with different antigen proteins according to different antibody groups. Reactions with each protein antigen were variable. Among S fragments, S3 reacted strongly in the high group.

In the S1 ELISA, the OD of the high group was 1.09–1.38, that of the medium group was 0.78–0.93, and that of the low group was 0.58–0.64 (Figure 5a). In the S2 ELISA, the OD of the high group was 1.34–1.47, that of the medium group was 1.21–1.35, and that of the low group was 0.33–0.44 (Figure 5b). In the S3 ELISA, the OD of the high group was 2.45–2.65, that of the medium group was 1.83–2.03, and that of the low group was 0.47–0.65 (Figure 5c). In the N pELISA, the OD of the high group was 2.88–2.95, that of the medium group was 2.13–2.62, and that of the low group was 1.87–2.02 (Figure 5d).

Based on the pELISA results, the OD values were correlated with the SNT. Interestingly, upon further analysis of the spike fragments, the S3 protein showed higher immunogenicity and induced higher neutralizing activity than the S1 and S2 fragments. This was the reverse of our speculation. Comparing the antibody titers of the spike and nucleocapsid proteins, the majority of antibody-generating vaccinations seem to protect against nucleocapsid proteins.

## 3. Discussion

PEDV mRNAs are transcribed and then translated to produce viral structural and nonstructural proteins [21]. Among structural proteins, spike protein (S) is important for specific receptor binding and cell membrane fusion. Thus, the S protein would be a target for the development of PEDV vaccines [22]. It is the dominant surface protein in all coronaviruses, has a role in specific receptor binding and cell membrane fusion. Spike proteins recognize cellular receptors via specific interactions and are responsible for mediating viral neutralizing antibodies [23,24]. The presence of serum antibodies against gastroenteric pathogens is not always correlated with protection; rather, detection of these antibodies only proves that individuals had contact with infectious microorganisms [25]. 

In this study, we evaluated sera from sows vaccinated against PEDV. We compared the IgG titer and SNT results and found a correlation. We also evaluated how many spike antibodies are generated by vaccination. There are many applied techniques in current practices, and attempts were made to explore the application of SNT and ELISA. Field serum samples (n = 62) were collected and screened to detect the presence of an antibody against PEDV using SNT and ELISA. We also performed pELISA to confirm which regions on the spike are more immunogenic. Unexpectedly, the protein expression of spike fragments S1 and S3 and nucleocapsid were slightly higher than that of S2, as shown in Figure 2. Although we loaded the same amount of protein on the SDS-PAGE gels, the Western blot signal was much lower than that of the other gels. Excluding technical errors, we speculate that the PEDV antibodies generated by vaccination did not recognize the S2 region or that S2 was not very immunogenic, so the number of antibodies generated against S2 was low; we still do not understand this matter clearly.

Correlated with the SNT results, samples with higher SNT titers showed higher IgG titers, and samples with lower SNT titers showed higher IgG titers (Figure 4). Our results confirm that there is a clear correlation between the SNT and ELISA titer. The purpose of the pELISA analysis was to confirm where neutralizing epitopes are located; for this purpose, we compared the OD values using S1, S2, S3 and N proteins as the coating antigen. In the pELISA, the antibodies against N were much higher than those against any of the spike protein fragments, as shown in Figure 5. Comparing only the spike fragments, the highest antibody titer was generated against the S3 fragment. This was the opposite result to our speculation. These results confirm that S3 might be a real neutralizing epitope, although it is a tail region of the PEDV spike protein. This is consistent with our earlier report [26]. In our report, we found that the neutralizing epitope was located at the tail region of the PEDV spike protein, characterized by the 1368GPRLQPY1374 motif [25]. We proved results in in vivo studies using synthetic peptides. There have been many reports on PEDV-neutralizing epitopes, and their locations differ. For example, the first reports were on the COE region; the COE epitope (a.a.422–638) neutralizing epitope was analyzed [26,27]. More recently, Li et al. reported that the cell attachment domain on the S1 region has key targets of neutralizing antibodies [28]. Kong et al. also reported the SE protein in the S1 region as the B cell epitope [29]. In contrast, Chang et al. reported that the N-terminus of the S2 region contains a neutralizing epitope [30].

We question how the carboxy terminal region generates neutralizing antibodies because it is located on the endodomain. Although it is known that the carboxy-terminal endodomain is involved in the assembly of the spike protein and progeny virions by interacting with the matrix protein, there can be an antigenic motif on the endodomain of a surface glycoprotein of viruses, such as the 746 ERDRD750 motif on HIV gp41 [31,32]. The exact membrane topology and location of this motif are still controversial, and it has been suggested that antibodies against this motif on the gp41 glycoprotein cross-react with an antigenically similar epitope on the ectodomain of the p17 matrix protein [30]. Additionally, importance of the GPRLQPY motif was proved by the strong antibody response against the peptide identical to the 24 amino acid tail region [33].

In summary, we demonstrated the correlation between the SNT and ELISA in PEDV-vaccinated sera. In addition, we confirmed neutralizing epitopes. Sera samples with higher neutralizing activity showed higher titers of IgG. Although we also performed ELISAs and pELISAs for IgA detection, the results were lacking, and we excluded the IgA results. In the antibody profiling results, neutralizing activities positively related with the spike antibody, especially the S3 region. We confirmed that the tail region induced stronger neutralizing activity. These results match with our previous findings [33]. We still do not know how the endodomain induces neutralizing antibodies and how this region is important for protection possibility. However, together with our previous results and results of this study, we could conclude that S3 protein could be useful for evaluating PED vaccine efficacy, and it is a strong neutralizing epitope of PEDV.

## 4. Materials and Methods

### 4.1. Sampling

Totally, 62 sera samples were collected from sows in swine farms located in different regions of Korea. Each farm has the same PED vaccination program under the biosecurity program. Five sera samples were collected randomly from each farm. Farms size and number of sows housed were different to each other. All collected sera were stored at –70 °C prior to use.

### 4.2. Viruses 

Vero cells were prepared in minimum essential medium supplemented with 5% fetal bovine serum (Gibco). The virus was propagated in Vero cells supplemented by MEM containing 0.001% trypsin (Invitrogen) at 37 °C, 5% CO_2_ for 48–60 h as described in a previous report with some modification such as adsorption and media change time [10]. 

### 4.3. RT-PCR for Amplification of Genes

For amplification of partial fragments of spike protein (S1, S2 and S3 locations described in Figure 1) and full nucleocapsid protein, RT-PCR was performed at 94 °C for 2 min, followed by 40 cycles of 94 °C 20 s, 54 °C 10 s, 72 °C 4 min, and a final extension at 72 °C for 10 min using primers as shown in Table 1. The amplified genes were subcloned into the corresponding restriction sites in pQE-30 (Qiagen, Hilden, Germany) and plasmids were then transformed into competent cell (DH5α) and confirmed.

### 4.4. Prokaryotic Expression

Briefly, the recombinant plasmids were transformed into *Escherichia coli* BL21 (DE3). Cells were harvested for 4 h for N and 5 h for S proteins after IPTG induction. Collected cells were washed with PBS three times and resuspended in lysis buffer [2.7 M sodium chloride, 54 mM potassium chloride, 86 mM sodium phosphate, 28 mM potassium phosphate buffer (pH 7.5) containing 100 mM PMSF (phenylmethylsulfonyl fluoride)].

### 4.5. SDS-PAGE and Immunoblot Analysis

The proteins were visualized in sodium dodecyl sulfate polyacrylamide gel electrophoresis (SDS-PAGE) as described by Laemmli [34]. Briefly, the expressed protein was separated by SDS-PAGE using 12% separating gel and 5% stacking gel. Gels were stained with Coomassie brilliant blue R250 (Acros Organics, Thermofisher Scientific, Waltham, MA, USA) and immunoblotting was performed according to the method of Burnette [12]. Briefly, separated proteins were electrically transferred onto a polyvinyl difluoride membrane and PEDV N protein was traced using anti-PEDV polyclonal antibodies. Bands were visualized using Supersignal West Dura (Thermofisher Scientific, Waltham, MA, USA) with LAS-1000PLUS.

### 4.6. Serum Neutralization Test (SNT)

The SNT was performed following the standard protocol routinely performed at our laboratory [15,28]. Briefly, Vero cells and viruses were maintained in minimal essential medium (MEM) supplemented with 10 % fetal bovine serum (FBS, Gibco, Thermofisher Scientific, Waltham, MA, USA), and 2% antibiotic–antimycotic agent mixture (Life Technologies, Carlsbad, CA, USA). All the sera were inactivated at 56 °C for 30 min and stored at –20 °C. Then, sera were diluted two-fold, and PEDV of 10^5^TCID_50_/mL was mixed with an equal volume of diluted sera. The mixture was incubated for 1 h at 37 °C. Subsequently, 0.1 mL of each virus–serum mixture was inoculated onto Vero cell monolayers, and the SNT values were given as number of times serum diluted versus serum gave 50% neutralization of PEDV.

### 4.7. Enzyme-Linked Immunosorbent Assay (ELISA)

The ELISA was performed following the conventional method. Briefly, the micro titration plates (SPL Life Sciences, Seoul, Korea) were coated with KPEDV-9 in bicarbonate buffer (pH 9.6) overnight at 4 °C. After 1st and 2nd antibodies treatments, TMB substrate (eBioscience, Thermofisher Scientific, Waltham, MA, USA) solution as chromogen was added into the wells. The plates were incubated for 10 min in the dark at room temperature. The color development was stopped by adding 50 µL/well of 2N H_2_SO_4_. The absorbance at 450 nm was measured and recorded for the statistics.

### 4.8. Protein ELISA (pELISA)

For protein ELISA, 96-well ELISA plates were coated with 100 µg/well of an equal mixture of each expressed protein diluted in 0.01 M carbonate buffer (pH 9.6). After overnight incubation at 4 °C. After anti-PEDV antibodies as the 1st and anti-swine IgG as the 2nd antibodies treatments, TMB substrate (eBioscience) solution as chromogen was added into the wells. The plates were incubated for 10 min in the dark at room temperature. The color development was stopped by adding 50 µL/well of 2N H_2_SO_4_. The absorbance at 450 nm was measured and recorded for the statistics.

## 5. Conclusions

We evaluated 62 sow sera samples for both SNT, ELISA and pELISA. Sera samples with higher neutralizing activity showed higher titers of IgG. In the antibody profiling results, neutralizing activities correlated with the levels of the spike antibody, especially the S3 region. We confirmed that the tail region of the S protein was important for neutralizing activity. These results match with our previous findings. We still do not know how S3 induces neutralizing antibodies and how this region is important for protection possibility. However, together with our previous results and results of this study, we could conclude that S3 protein could be useful for evaluating PED vaccine efficacy.

## Figures and Tables

**Table 1:** The primer and sequence of S1, S2, S3, and nucleocapsid proteins.

Proteins (Gene Size)	Primer	Sequence
**Spike S1** (1450 bp)	PED S1-S	5′ GGA TCC ATG AGG TCT TTA ATT TAC TTC 3′
	PED S1-AS	5′ GGT ACC ATG GGG TAAA AAC CAT CGT CAA GGT CAA AAG 3′
**Spike S2** (1499 bp)	PED S2-S	5′ GGA TCC TTA CCC CAT CTC TTC TAG AAA CCT 3′
	PED S2-AS	5′ GGT ACC TAC AAA CAT ATG TAG CAC AAT CAA CAA CAC AC 3′
**Spike S3** (1469 bp)	PED S3-S	5′ GGA TCC AAG ACT TGC TTT TAA ATA 3′
	PED S3-AS	5′ GGT ACC TTA GTG ATG GTG ATG GTG ATG CTG CAC GTG AAC CT3′
**Nucleocapsid** (1326 bp)	PED N-S	5′ GGA TTC GCA TCT GTC AGC TTT CAG 3′
	PED N-AS	5′ GGT ACC TTA ATT TCC TGT ATC GAA 3′

## Data Availability

Not applicable.
